# ECG data dependency for atrial fibrillation detection based on residual networks

**DOI:** 10.1038/s41598-021-97308-1

**Published:** 2021-09-14

**Authors:** Hyo-Chang Seo, Seok Oh, Hyunbin Kim, Segyeong Joo

**Affiliations:** Department of Biomedical Engineering, Asan Medical Institute of Convergence Science and Technology, Asan Medical Center, University of Ulsan College of Medicine, Seoul, Republic of Korea

**Keywords:** Atrial fibrillation, Computer science

## Abstract

Atrial fibrillation (AF) is an arrhythmia that can cause blood clot and may lead to stroke and heart failure. To detect AF, deep learning-based detection algorithms have recently been developed. However, deep learning models were often trained with limited datasets and were evaluated within the same datasets, which makes their performance generally drops on the external datasets, known as data dependency. For this study, three different databases from PhysioNet were used to investigate the data dependency of deep learning-based AF detection algorithm using the residual neural network (Resnet). Resnet 18, 34, 50 and 152 model were trained with raw electrocardiogram (ECG) signal extracted from independent database. The highest accuracy was about 98–99% which is evaluation results of test dataset from the own database. On the other hand, the lowest accuracy was about 53–92% which was evaluation results of the external dataset extracted from different source. There are data dependency according to the train dataset and the test dataset. However, the data dependency decreased as a large amount of train data.

## Introduction

Atrial fibrillation (AF) is the most common cardiac arrhythmia which is irregular or rapid heartbeat. The number of AF patients is expected to increase by 12.1 million^1^ and the related cost of AF is estimated at USD 6–26 billion per year^2^ in US. Furthermore, AF can not only form thrombosis, which is the can cause stroke, but also affect heart failure and other heart disease^3^. AF rises the risk of stroke five times^4^ and the risk of death twice^5^, compared to healthy a person. Therefore, considering the social cost of healthcare and the quality of life, early and accurate detection of AF is important and beneficial. In the clinical environment, the detection of AF is manually done with visual inspection of the electrocardiogram (ECG) recordings. Cardiologists inspect the ECG recordings collected about 24 h by ambulatory ECG device (Holter monitor). However, manually inspecting large amounts of ECG recordings can be tedious and time-consuming^6,7^. Also, time and frequency components of ECG are very subtle for accurate and consistent manual inspection^7^. A study showed that the manual inspections of many primary care practitioners are insufficient for accurate detection of AF^8^. This implies that there are limitations in detecting hidden patterns of AF and extensive training of clinician is necessary to find AF effectively.

Recently, with the emerging research on artificial intelligence (AI), automatic AF detection algorithms have been developed to resolve above problems. Reported AI based AF detection algorithms generally utilizes machine learning or deep learning techniques. Machine learning based AF detection algorithms employ features, which are measured or calculated by original ECG signal^9–17^. This feature extraction step is important for the machine learning based AF detection algorithms. However, it is generally the most time-consuming process in developing those algorithms. Recent year, deep learning-based AF detection algorithms have been developed. Deep learning is an AI algorithm that automatically train the computational model to solve complex problems. Model learns a representation of the data through training the multiple processing layers. Afterward, this trained model can be used to predict events on new data with performance beyond human-level. Due to these advantages, deep learning techniques is widely used nowadays in various healthcare applications such as medical imaging, drug discovery, and genomics.

However, with the high performance of the most deep learning-based algorithm developed in healthcare field, they suffer from data dependency, which means that the developed algorithm generally works well within the database used for the development but the performance generally drops when the algorithm was used in other database. Unlike general applications of deep learning, healthcare data is highly heterogeneous, ambiguous, noisy, and incomplete. Furthermore, healthcare data collected from different medical institutions, hospitals, or devices is uneven and no uniform which can lead to worthless analysis^18^. To avoid adverse effect on patient, thorough validation is necessary before applying deep learning-based algorithm to healthcare data. The validation using external data collected from various devices or institutions is important to evaluate the generalization performance of deep learning-based algorithm. However, deep learning-based algorithm is generally validated by the internal database used for the development. For example, in the medical imaging application included radiology, ophthalmology, and pathology diagnostic analysis, most deep learning-based algorithms did not employ the validation using external database^19^.

Deep learning model build with AF data collected from the different setting, such as sampling frequency, resolution, and acquisition environment, may suffer from data dependency. There are several open databases for studying heart related research. Many previously reported papers for making AF detection algorithm utilized these databases. However, most research does not consider the data dependency, which can be problem when the algorithms are used in real environment. In this study, to quantity this data dependency, we experimentally investigated the data dependency of deep learning model of AF classification build with those open databases.

## Methods

**Figure 1:**
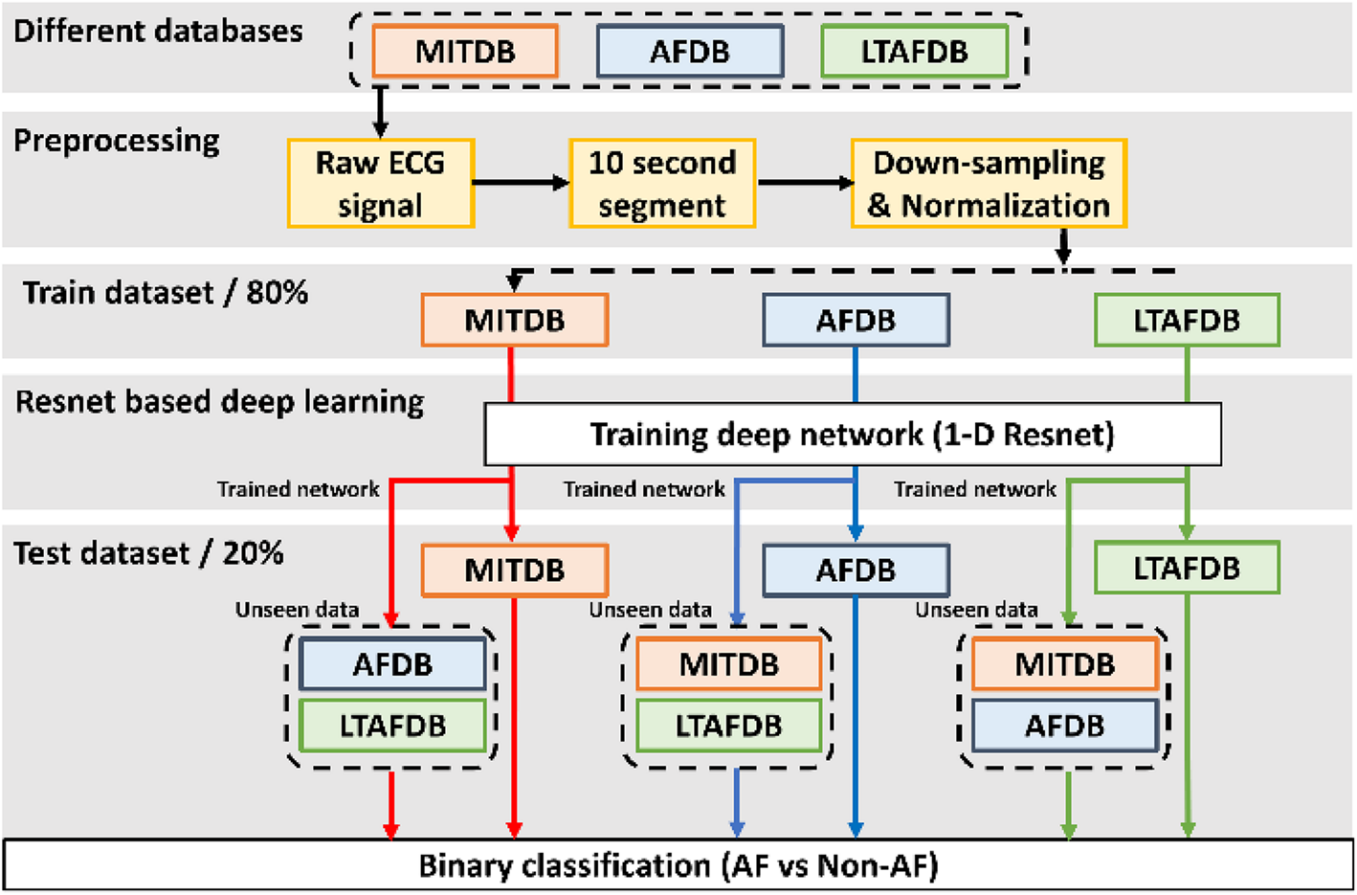
Overview of our study.

We train the deep learning model using three different AF databases and evaluate the data dependency using not used for training. The training method is described in Fig. 1.

### Open database

The three open databases on Physionet, Long-Term Atrial Fibrillation database (LTAFDB)^20^, MIT-BIH Atrial Fibrillation database (AFDB)^21^, MIT-BIH Arrhythmia database (MITDB), are used^22^. The LTAFDB consist of 84 subjects with paroxysmal or sustained atrial fibrillation, which is two-channel ECG signal digitized at 128 Hz with 12-bit resolution over 20 mV range for about 24–25 h^20^. The annotated diseases are normal sinus rhythm (N), supraventricular tachyarrhythmia (SVTA), ventricular tachycardia (VT), atrial fibrillation (AF), ventricular bigeminy (B), ventricular trigeminy (T), idioventricular rhythm (IVR), and atrial bigeminy (AB), sinus bradycardia (SBR). The AFDB is composed of 25 subjects with atrial fibrillation (mostly paroxysmal), which is two-channel ECG signals each sampled at 250 samples per second with 12-bit resolution over a range ± 10 mV for 10 h. The rhythm annotation files were prepared manually. The rhythm annotations of types are atrial fibrillation (AF), atrial flutter (AFL), atrioventricular junctional rhythm (J), and other rhythms (N)^21^. In this study, two ECG recordings of AFDB (records 00735 and records 03665) were excluded because they are unavailable. The MITDB with 48 half-hour two-channel ECG recordings are included in 47 subjects. The 23 recordings were collected from a mixed population of inpatient (about 60%) and outpatient (about 40%). The remaining 25 recordings were collected from the same set to include less common but clinically significant arrhythmias. The recordings were digitized at 360 samples per second per channel with 11-bit resolution over a 10 mV range^22^. In the LTAFDB and MITDB, we used AF rhythm as “AF” class and normal sinus rhythm as “Non-AF”. In the AFDB, since there is no normal sinus rhythm annotation, we used AF type rhythm as “AF” class and other rhythms type rhythm as “Non-AF” class. All the two-channel ECG signals in each database are used to training and test datasets. The detailed descriptions about the databases are shown on Table 1.

**Table 1:** Description of three different databases, LTAFDB, AFDB, and MITDB.

	LTAFDB	AFDB	MITDB
No. of recording	84 records	25 records	48 records
Channel	2 Ch	2 Ch	2 Ch
Duration	24–25 h	10 h	Half-hour
Sapling rate	128 Hz	250 Hz	360 Hz
Resolution	12 bit	12 bit	11 bit
Voltage range	20 mV	± 10 mV	10 mV
Acquisition location	Not reported	Boston’s Beth Israel Hospital	Boston’s Beth Israel Hospital

### AF detection model

**Figure 2:**
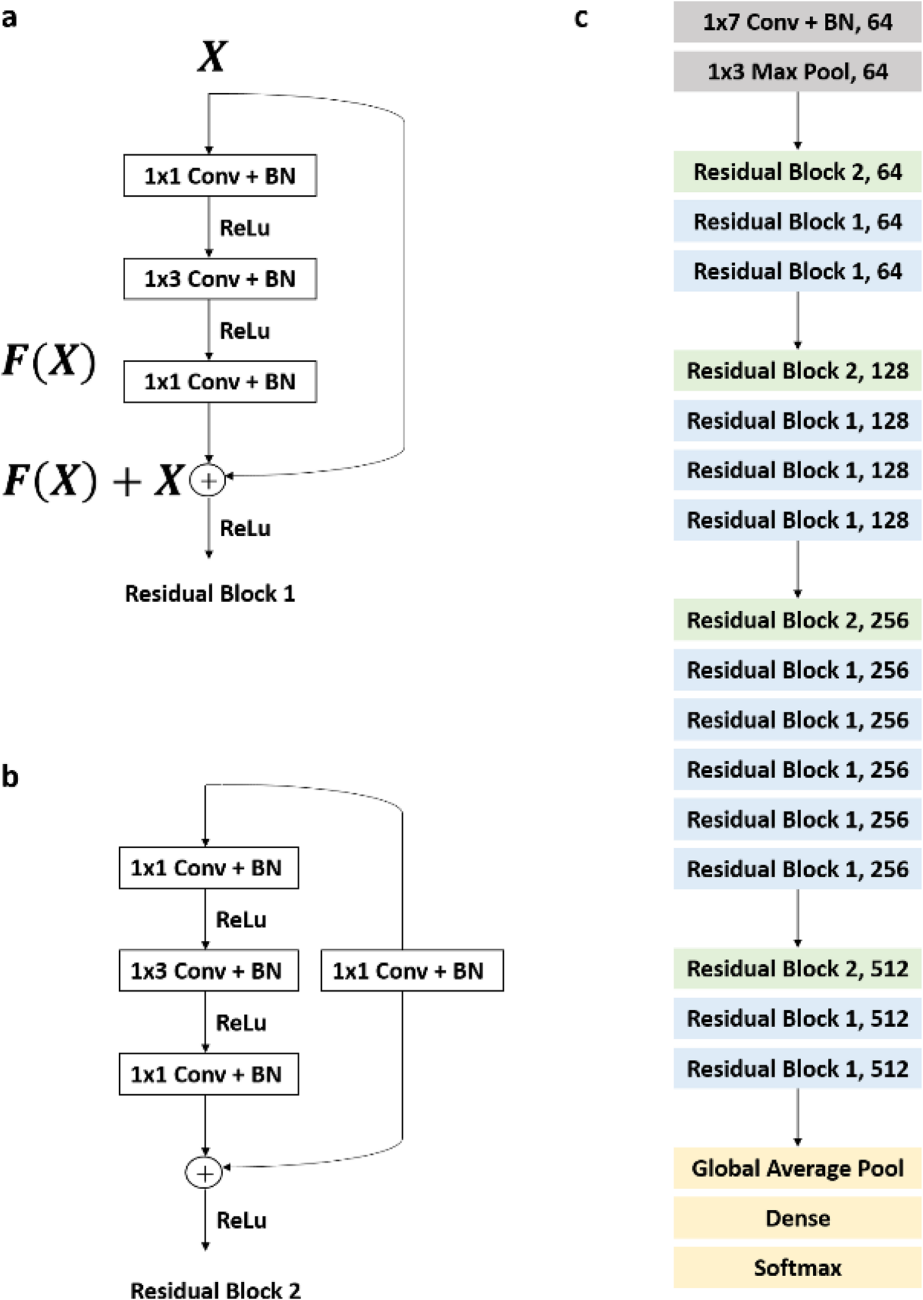
Schematic diagram of residual block. (**a**) Residual block when previous layer and present layer are same dimensions. (**b**) Residual block when previous layer and present layer are different dimensions. (**c**) Resnet 50 architecture.

To maintain data independence, the group of patients was separated into train and test dataset, and then the data was divided into 10-s segments. The number of ECG segments used for experiments is listed in Table 2. Since the sampling rate of the LTAFDB, the AFDB and the MITDB are different as 128 Hz, 250 Hz and 360 Hz respectively, the AFDB and MITDB are downsampled at 128 Hz. In the addition, Each ECG data are normalized by Z-score normalization. The normalized ECG data are divided into a duration of 10 s (1280 samples) for input size. The residual network (Resnet) model developed by He^23^ is used for AF detection because this Resnet model has recently been used for a lot of studies on cardiac arrhythmia classification^24–26^. Resnet has a good performance without gradient vanishing because of the shortcut of the previous layer X to layer ahead F(X) as shown in Fig. 2a. If the dimension of X and F(X) are not match, the convolution layer (Conv) and Batch Normalization (BN) are used to match the spatial resolution as shown in Fig. 2b. In this study, we employed different 1-D Resnet models to detect AF. The architecture of original Resnet model is converted 2-D to 1-D for training 1-D ECG signal. Additionally, we trained the 1-D Resnet 18, 34, 50, and 152 layers at each three database and compared with each other. For instance, Resnet 50 architecture as shown in Fig. 2c. We used the cross-entropy which is well known cost function on classification problem. Subsequently, the cost function was minimized by Stochastic Gradient Descent optimizer. The initial learning rate was set to 0.001. A momentum was set to 0.9 and a weight decay set to 0.0001 based on^23^. Mini-batch was size of 32^27^. The learning rate was divided by 10 when error plateaus, and the weight of networks was initialized as in^28^. The early stopping technique was implemented over 10 epochs to avoid overfitting. We train and test different Resnet with 18, 34, 50, and 152 layers composed of training dataset 80% and test dataset 20% using LTAFDB, AFDB, and MITDB, independently. We separated a test set and a training set for each individual to verify independence, and used 20% of the training set as a validation set to check performance.

**Table 2:** The number of data segments for AF classification.

	Train data		Test data		Total data	
	Non-AF	AF	Non-AF	AF	Non-AF	AF
LTAFDB	460,740	588,990	115,615	146,816	576,355	735,806
AFDB	79,960	53,535	19,944	13,428	99,904	66,963
MITDB	9696	1213	2435	291	12,131	1504
NSRDB	-	-	314,982	-	314,982	-

### Performance evaluation

The confusion matrix visualizes the summary of classification results and reports the number of true positive, true negative, false positive and false negative. The confusion matrix is used to visualize the performance of trained model datasets composed of independent databases. The Receiver Operating Characteristic curve (ROC curve) illustrate the Sensitivity against the false positive rate for various decision thresholds. The Area Under Curve (AUC) is a populate evaluation metric which measures the area under entire ROC curve. We used the ROC curve and AUC to present the data dependency according to dataset. Finally, statistics is used to report the data dependency according to different Resnet models (Resnet 18, 34, 50 and 152). They are accuracy defined as the proportion of correctly classified segments among the total number of segments, sensitivity defined as the proportion of true positive among the total number of positive segments, and specificity defined as the proportion of true negative among the total number of negative segments.

## Results

The experiments were executed on python package with Keras, along with the computer environments of Intel(R) Core(TM) i7-6900 k CPU 3.20 GHz, NVIDIA Geforce GTX 1080 Ti of GPU and Windows 10 operating system.

We evaluated different Resnet models (Resnet 18, 34, 50 and 152) as shown in Table 3. In each trained model, the highest accuracy is about 98–99% on the test dataset of internal database. On the other hand, the lowest accuracy is about 53–92% on the test dataset of external database. Initially, the differences between highest and lowest accuracy in Resnet 18 model are 17.26% on the training model of LTAFDB, 18.87% on the AFDB and 44.59% on the MITDB, respectively. Secondly, those of Resnet 34 model are 17.97% on the LTAFDB, 21.24% on the AFDB and 44.10% on the MITDB, respectively. Those of Resnet 50 model are 16.90% on the LTAFDB, 20.44% on the AFDB and 45.89% on the MITDB. Those of Resnet 152 model are 16.42% on the LTAFDB, 19.34% on the AFDB and 42.92% on the MITDB. There is no significant performance difference according to the number of layer, so that following experiments were executed with Resnet 50 model which has medium depth in the models used in the experiment.

**Table 3:** Performance results of different Resnet models.

Trained model		Test data		
Model	Train data	LTAFDB	AFDB	MITDB
Resnet 18	LTAFDB	**99.04**	92.03	82.10
	AFDB	85.01	**99.41**	86.68
	MITDB	74.48	70.98	**99.89**
Resnet 34	LTAFDB	**98.70**	92.19	81.14
	AFDB	84.94	**99.27**	78.14
	MITDB	63.65	65.55	**99.78**
Resnet 50	LTAFDB	**98.66**	92.13	83.90
	AFDB	84.35	**99.20**	80.48
	MITDB	68.79	65.61	**99.82**
Resnet 152	LTAFDB	**98.53**	92.00	84.67
	AFDB	83.87	**99.21**	81.14
	MITDB	66.23	64.41	**99.56**

**Figure 3:**
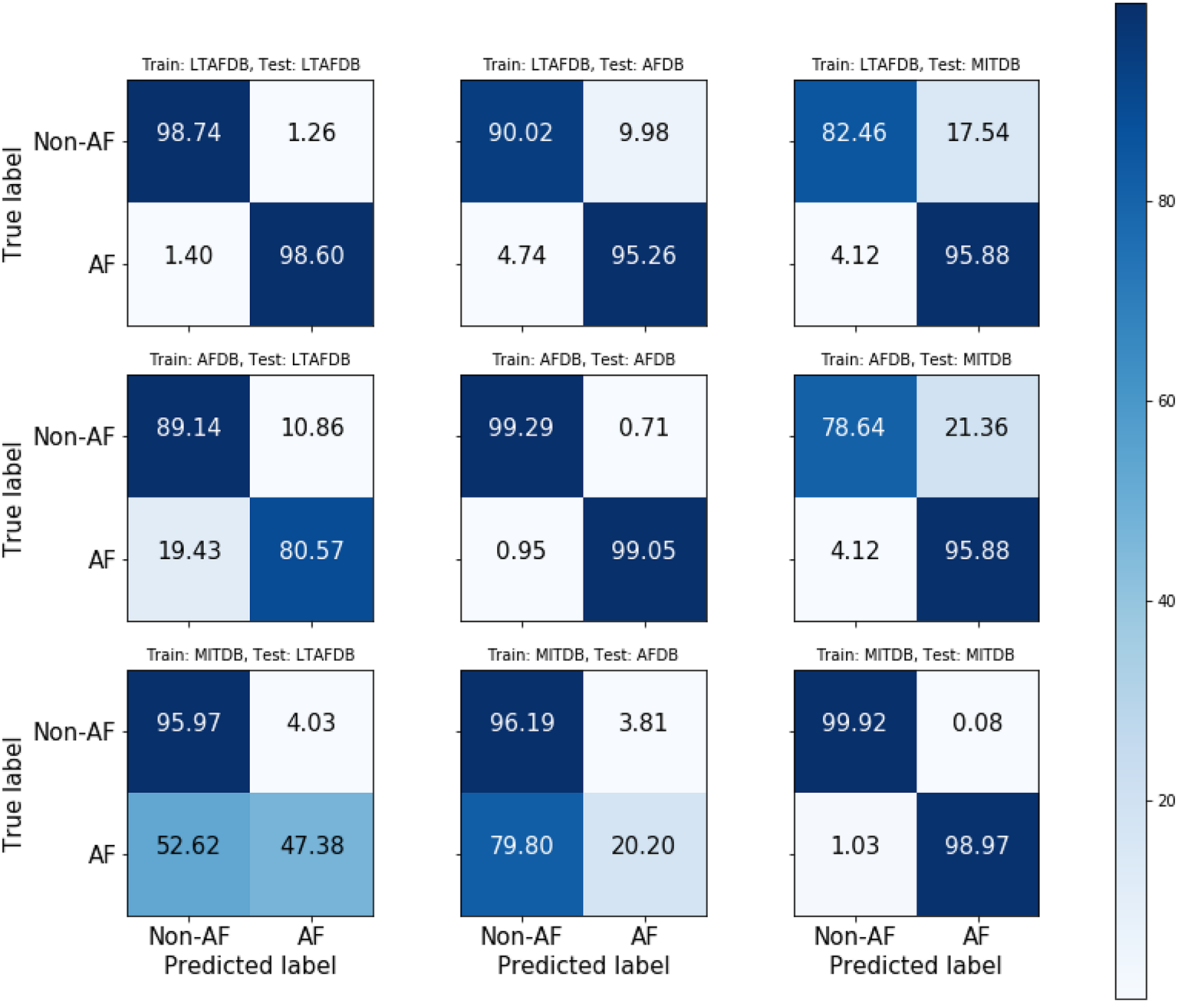
Confusion matrix of the Resnet 50 model for estimating data dependency. The confusion matrices in the first row are results of the trained models on LTAFDB. The second row shows results of the trained models on AFDB and the third row shows results of the trained models on MITDB. The confusion matrices in the first column are results evaluated by LTAFDB. Also, the second column show results evaluated by AFDB and the third column show results evaluated by MITDB.

The confusion matrices of Resnet 50 model are represented Fig. 3. In the case of the trained model on LTAFDB, the evaluated case by internal database (LTAFDB) shows the highest true positive rate 98.76% and true negative rate 98.55%. However, the highest false negative rate 5.60% is reported on the evaluated case by the external database (AFDB) and the highest false positive rate 20.23% is reported on the evaluated case by the external database (MITDB). Secondly, in the case of the trained model on AFDB, the highest true positive rate 99.11% and true negative rate 99.43% are resulted on the evaluated case by the internal database (AFDB). However, the highest false negative rate 33.12% is resulted on the evaluated case by the external database (LTAFDB) and the highest false positive rate 21.26% is resulted on the evaluated case by the external database (MITDB). Thirdly, in the case of the trained model on MITDB, the highest true positive rate 98.27% and true negative rate 99.84% are resulted on the evaluated case by the internal database (MITDB). However, the highest false negative rate 76.86% and false positive rate 7.03% are resulted on the evaluated case by the external database (AFDB).

**Figure 4:**
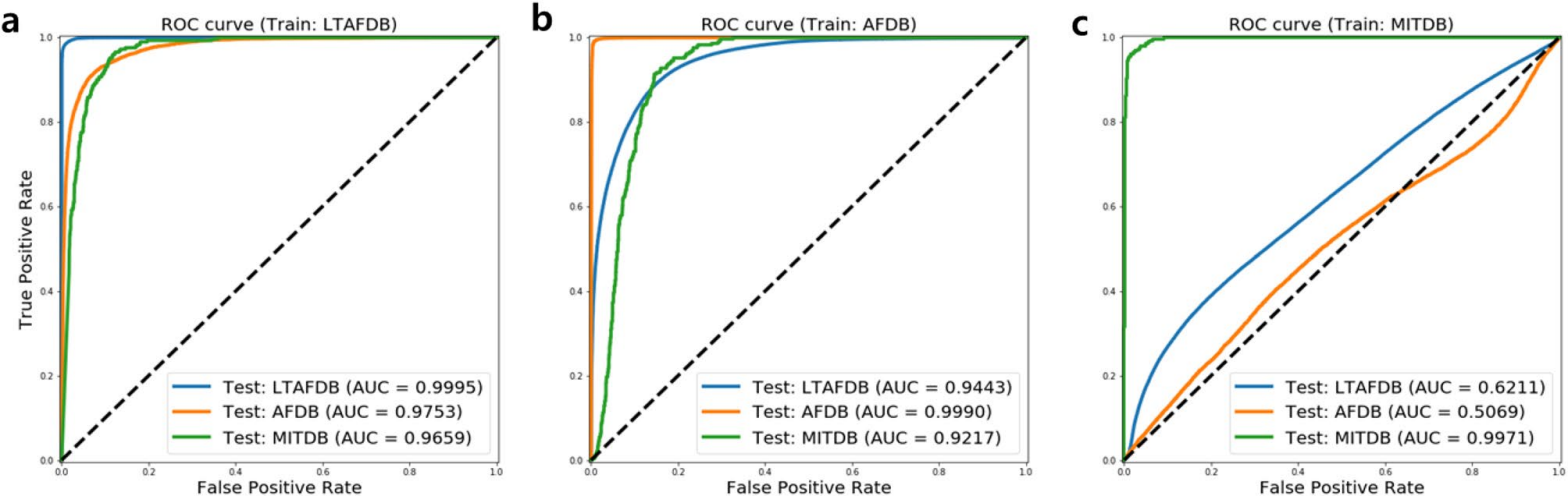
ROC curve of the Resnet 50 model for estimating data dependency. (**a**) The results of train model on LTAFDB. (**b**) The results of train model on AFDB. (**c**) The results of train model on MITDB.

Next, the ROC curve of Resnet 50 model is shown as Fig. 4. Initially, Fig. 4a shows the ROC curves of trained model on LTAFDB. The highest AUC score is 0.9994 on the evaluated case by the internal database (LTAFDB) and the lowest AUC score is 0.9494 on the evaluated case by external database (MITDB). Figure 4b shows the results of trained model on AFDB. The highest AUC score is 0.9993 on the evaluated case by internal database (AFDB) and the lowest AUC score is 0.9190 on the evaluated case byexternal database (MITDB). Figure 4c shows the results of trained model on MITDB. The highest AUC score is 0.9999 on the evaluated case by the internal database (MITDB) and the lowest AUC score is 0.5296 on the evaluated case by the external database (AFDB).

In order to estimate the data dependency only healthy subjects, we evaluated the trained models on MIT-BIH Normal Sinus Rhythm database (NSRDB)^29^ composed to only normal sinus rhythm (“Non-AF” class) recorded from patients had no significant arrhythmias. It is good estimate of the false positive rate in healthy subjects^30^. The trained 50 models on LTAFDB, AFDB and MITDB were performed with the specificity of 97.16, 97.60, and 95.46 and false positive rate of 2.84, 2.40 and 4.55, respectively. It is listed in Table 4.

**Table 4:** Results of the Resnet 50 model on NSRDB.

Train data	NSRDB	
	Sp (%)	Fpr (%)
LTAFDB	97.16	2.84
AFDB	97.60	2.40
MITDB	95.45	4.55

## Discussion

We experimentally investigate the data dependency of deep learning-based AF classification using Resnet and raw ECG signal. As indicated in Table 3, the highest accuracy of each trained model was resulted on evaluating the test dataset extracted from own database within all Resnet model used in this study (Resnet 18, 34, 50 and 152). In contrast, the model accuracy was decreased on evaluating the external dataset extracted from different source. Resnet generally shows a good performance without gradient exploding or gradient vanishing even if model is much deeper network. However, the data dependency occurs regardless of the depth in Resnet architecture. Therefore, the deeper network cannot resolve the data dependency. On the unseen data, when the true positive rate increases, the false positive rate also tends to occur higher. Also, the true negative rate and false negative rate also show the same trend. Unlike the evaluation results of own database, if model show a high sensitivity for external data, specificity oppositely is low. Similarly, the high specificity for external data lead to low sensitivity in trained models. These results imply that the trained model may biasedly predicts the external data to be positive or negative.

In this study, although dependency is a widely known and verified phenomenon specially in medical imaging, it is meaningful that we have proven this phenomenon to the first time in 1-D signals such as bio-signals. It was performed using Resnet, which is the most widely used ECG classification algorithm, but it do not solved dependency problem. Various normalizing methods were tried, while the dependency could not be perfectly removed.

When evaluate the external data extracted from NSRDB, all trained Resnet 50 models with the LTAFDB, AFDB, and MITDB showed specificity more than 95% and false positive rate about 2–4%. The specificities of the trained models tested with NSRDB was higher than that of the trained models with other databases except the database used for building the model. These results implies that normal sinus rhythm of healthy patients less suffers from data dependency.

The data dependency in building AI models can be caused by several aspects. Data imbalance is one of the most common cause. If a database has AF events far lesser than normal rhythms, this is common in medical databases in general, the performance can be biased and the performance can be drop when tested with external database. Another problem is noise in ECG signals by motion artifact or other reasons. Physical movement of patient when measuring ECG can cause wandering of baseline of ECG or unwanted noises. These noises can be minimized from digital filtering or other signal processing. However, during these processes, distortion or losing characteristic waveform of the original ECG can occur and the processed signals can have different characteristics according to the method of the preprocessing. The performance of AI models can lower due to the difference in preprocessing method of the database. The other problem is discrepancy in measuring hardware. There are several companies making devices for measuring ECG. These devices have different in hardware settings, such as amplifier configuration, filters, and gain, and software settings, such as sampling frequency and resolution. The waveforms from approved ECG devices do not differ largely, and resampling or normalization technique can reduce these problems but not perfectly resolve.

It can be concluded that it is necessary to validate the deep learning based AF detection algorithm using the various external databases at the developing step to avoid the data dependency.

### Limitation

There are some limitations in this study. Initially, this study was implemented with only one deep learning architecture, Resnet. Evaluation using other deep learning architectures will be helpful in investigating the data dependency. Secondly, “Non-AF” classes in the MITDB and LTAFDB are composed of normal sinus rhythm but “Non-AF” class in the AFDB is composed of all other rhythm because of the absence of normal sinus rhythm annotation. This limitation may lead to low specificity when evaluate the AFDB. However, the performance of sensitivity could be effectively reflected. Thirdly, the data used in this study is from three open-source databases, LTAFDB, AFDB, and MITDB. The using more databases collected from various location, device, and setting will be helpful to effective research results.

The MITDB has the imbalanced and smallest amount of data among the databases used in this study. The trained models of those show a largest data dependency in the experiment results. On the contrary, the LTAFDB has the largest amount of data among them. In the trained model with LTAFDB, the data dependency is lower than other trained models. Also, these results imply that training the deep learning model using the large amount and balanced data can decrease the data dependency on the AF detection algorithm. However, the acquisition and use of large amount AF data may be difficult because of the patient privacy and legal issues for healthcare data.

## Data Availability

Open database used in this study is illustrated in Methods.
